# Trait Self-Compassion Reflects Emotional Flexibility Through an Association with High Vagally Mediated Heart Rate Variability

**DOI:** 10.1007/s12671-016-0549-1

**Published:** 2016-06-02

**Authors:** Julie Lillebostad Svendsen, Berge Osnes, Per-Einar Binder, Ingrid Dundas, Endre Visted, Helge Nordby, Elisabeth Schanche, Lin Sørensen

**Affiliations:** 1 Department of Biological and Medical Psychology, Faculty of Psychology, University of Bergen, Jonas Lies vei 91, Bergen, 5009 Norway; 2 Bjørgvin District Psychiatic Centre, Knarvik, Haukeland University Hospital, Kvassnesvegen 63, 5914 Isdalstø, Norway; 3 Department of Clinical Psychology, Faculty of Psychology, University of Bergen, Christiesgate 12, Bergen, 5015 Norway; 4 KG Jebsen Center for Neuropsychiatric Disorders, Bergen, Norway

**Keywords:** Self-compassion, Heart rate variability, Emotion regulation, Emotional flexibility, Young adults

## Abstract

Converging evidence shows a positive effect of self-compassion on self-reported well-being and mental health. However, few studies have examined the relation between self-compassion and psychophysiological measures. In the present study, we therefore examined the relation between trait self-compassion and vagally mediated heart rate variability (vmHRV) in 53 students (39 female, mean age = 23.63). Trait self-compassion was assessed using the Self-Compassion Scale, and resting vmHRV was measured during a 5-min ECG baseline period. We hypothesized that higher levels of trait self-compassion would predict higher levels of resting vmHRV. Controlling for potential covariates (including age, gender, and BMI), the results confirmed our hypotheses, showing that higher levels of trait self-compassion predicted higher vmHRV. These results were validated with a 24-h measure of vmHRV, acquired from a subsample of the participants (*n* = 26, 16 female, mean age = 23.85), confirming the positive correlation between high trait self-compassion and higher vmHRV. The relation between trait self-compassion, vmHRV, self-reported trait anxiety (the trait scale of the State-Trait Anxiety Inventory; STAI) and self-reported rumination (the Rumination subscale of the Rumination-Reflection Questionnaire; RRQ-Rum) was also investigated. Higher levels of trait anxiety and rumination were highly correlated with low levels of trait self-compassion. Trait anxiety, but not rumination, correlated marginally significantly with the level of vmHRV. The findings of the present study indicate that trait self-compassion predicts a better ability to physiologically and psychologically adapt emotional responses. Possible implications and limitations of the study are discussed.

**Electronic supplementary material:**

The online version of this article (doi:10.1007/s12671-016-0549-1) contains supplementary material, which is available to authorized users.

## Introduction

Self-compassion—the ability to be kind and caring toward oneself in times of suffering—is found to be positively associated with mental health and well-being (e.g., Hollis-Walker and Colosimo 2011; Neff 2003b; Neff et al. 2007b). Although there are strong reasons to expect a positive association with physiological health as well, there is a lack of studies examining the relation between self-compassion and physiological indices such as vagally mediated heart rate variability (vmHRV). High vmHRV is an index of healthy heart function and is suggested as a physiological index of emotion regulation capacity, reflecting an ability to effectively adapt to stress and environmental demands (Appelhans and Luecken 2006; Beevers et al. 2011; Thayer and Lane 2000). As self-compassion seems to be an effective emotion regulation strategy (Neff 2003b; Neff et al. 2005), we predicted in the current study that high trait self-compassion is related to high vmHRV.

Emotion regulation is the processes through which one shapes which emotions one has, when one has them, and how one experiences or expresses them (Gross 1998, 2014). Trait self-compassion can be viewed as an implicit (predominantly unconscious and autonomic) strategy of emotion regulation in making an individual more accepting and less judgmental toward possible intrinsic and extrinsic stressors. It comprises three pairs of opposing components: (a) self-kindness vs. self-judgment, which is the tendency to be kind and understanding toward oneself in times of suffering, instead of critical and self-blaming; (b) common humanity vs. isolation, which refers to the recognition that imperfection and failure are normal parts of life, as opposed to feeling separated and disconnected from other individuals in difficult times; and (c) mindfulness vs. over-identification, which entails holding painful emotions and thoughts in balanced awareness, instead of avoiding, suppressing or over-identifying with them (Neff 2003a, see Table 1).

**Table 1:** Overview of the components of self-compassion

Components of self-compassion		
(a) Self-kindness	Self-kindness	The ability to treat oneself kindly and with compassion when one is challenged by suffering, failure, or difficult situations
	vs.	
	Self-judgment	Criticizing or judging oneself for one’s suffering
(b) Common humanity	Common humanity	Recognizing that suffering is part of being human
	vs.	
	Isolation	Feeling isolated and alone in one’s suffering
(c) Mindfulness	Mindfulness	Holding the experience suffering in non-judgmental awareness
	vs.	
	Over-identification	Over-identifying with the feelings of suffering

The role of self-compassion as an emotion regulation strategy is illustrated in a large body of research. Self-compassion has been found to moderate negative emotions after receiving ambivalent feedback, and to predict a lower level of negative emotions when experiencing difficult everyday situations (Leary et al. 2007). Thus, it seems to make individuals more emotionally flexible when experiencing negative life events, which is also evidenced in a reduced risk of depression (e.g., MacBeth and Gumley 2012; Raes 2011; Van Dam et al. 2011), less anxiety (Neff 2003b), and less rumination and thought suppression (Neff and Vonk 2009). Self-compassion has been proposed as a key mechanism of change in the positive effects of mindfulness-based cognitive therapy (MBCT; Kuyken et al. 2010), and high self-compassion is found to associate with happiness and optimism (Neff et al. 2007b), higher psychological well-being (Hollis-Walker and Colosimo 2011), and greater life satisfaction (Neff 2003b; Neff and Faso 2015). In fact, self-compassion remains positively correlated with overall positive affect even when controlling for high self-esteem (Neff and Vonk 2009), indicating that it contributes uniquely to feelings of worth and acceptance (Barnard and Curry 2011).

The positive effect of self-compassion is expected to have a soothing effect on heart rate and potential stress reactions. Inner self-talk that has a caring and supportive quality is thought to have similar effects as early attachment experiences of being soothed by significant caregivers through stimulating the mammalian oxytocin-opiate caregiving system (Gilbert 2009). Hence self-compassion is linked to feelings of being safe, because one knows that although things go wrong or one makes a mistake, one will not be met by harsh self-condemnation. When individuals feel safe, they tend to have a larger behavioral flexibility in different situations (Gilbert 2009), as well as higher physiological flexibility (Thayer and Lane 2000). Self-compassion is therefore likely to have a positive influence on the level of HRV, an index of physiological flexibility, defined as the variation in the time intervals between subsequent heartbeats (Appelhans and Luecken 2006; Thayer and Lane 2000). As the brain’s integrative system for emotion regulation has an inhibitory effect on the heart through the vagus nerve (Thayer and Lane 2000), we expect self-compassion to associate with high vagally mediated HRV (vmHRV; Williams et al. 2015).High vmHRV indicates increased parasympathetic influence on the heart (Appelhans and Luecken 2006; Thayer and Lane 2000) and is linked to a better ability to self-soothe when stressed (Porges 2007). Self-compassion is thus expected to lead to more flexible emotional and physiological responses, calming potential stress reactions by suppressing sympathetic activity and increasing parasympathetic influence through the stimulation of the vagus nerve (Porges 2003).

There are three lines of evidence indicating that self-compassion relates to higher vmHRV as well as to other biological processes underlying emotion regulation and stress responding. First, emotional stress responses such as anxiety (Chalmers et al. 2014; Thayer et al. 1996), depression (Beevers et al. 2011; Yeh et al. 2016), and rumination (Brosschot et al. 2007; Woody et al. 2014) are consistently shown to associate with lower vmHRV. Self-compassion correlates strongly with lower levels of such stress responses (e.g., Krieger et al. 2013; MacBeth and Gumley 2012) and therefore self-compassion is likely to associate with higher vmHRV. Second, studies examining self-compassion as a state and trained ability have found links with vmHRV. One study (Arch et al. 2014) found that participants who had received self-compassion training had smaller reductions in vmHRV before and after exposure to a psychosocial stressor as compared to two control groups. In another study (Rockliff et al. 2008), imagining receiving compassion associated with elevated vmHRV levels in some participants, and a more threat-like response in other participants. There were some indications that self-compassion affected the results, and the participants that responded with increased vmHRV also had a drop in cortisol levels. Another study (Kok et al. 2013) found compassion meditation to result in increased vmHRV. Third, self-compassion also seems to relate positively to other biological stress indexes than vmHRV. A study (Pace et al. 2009) measuring the effect of a 6-week compassion meditation training found a correlation between amount of meditation practice and decrease in the blood inflammation cytocine interleukin-6 after a psychosocial stressor. The group that received brief self-compassion training also had lower salivary alpha-amylase concentration, another marker of autonomic nervous system activation (Rohleder et al. 2004). Two recent studies (Breines et al. 2015; Breines et al. 2014) focused on trait rather than induced self-compassion, in contrast to the other studies. They found that participants higher in trait self-compassion had lower increases in blood inflammation (interleukin-6; Breines et al. 2014) and salivary alpha-amylase concentration (Breines et al. 2015) after a psychosocial stressor. Together these studies indicate that self-compassion may protect against physiological stress responses.

On the basis of this converging evidence of a positive association between self-compassion and mental and physical health, we wanted to investigate the relationship between trait self-compassion and vmHRV. To our knowledge, no prior studies have examined this relationship. We expected higher levels of trait self-compassion in a group of young healthy adults to predict higher levels of vmHRV. We further expected trait self-compassion to reflect lower trait anxiety and negative rumination. Since a large body of evidence supports the relationship between self-compassion and reduced levels of anxiety and rumination (e.g., MacBeth and Gumley 2012; Neff and Vonk 2009), and as trait anxiety and rumination also have been shown to associate with vmHRV (e.g., Williams et al. 2015; Woody et al. 2014), we also wanted to take into account trait anxiety and rumination when investigating the effect of self-compassion on vmHRV. We thus expected that the prediction of high trait self-compassion on vmHRV would sustain even when controlling for higher levels of trait anxiety and rumination. Finally, to validate that the predictive effect of higher levels of trait self-compassion on resting vmHRV was representative for the participants’ everyday lives, we also expected to find that higher levels of trait self-compassion would predict a higher level of a 24-h measure of vmHRV in a subsample of the healthy adults included in our study. Throughout this article, the term vmHRV will be used to refer to the 5-min resting condition, whereas the term 24-h vmHRV will be employed when referring to the 24-h measure.

## Method

### Participants

Data presented in the current paper were collected from a pilot study for a larger study on the effects of mindfulness-based cognitive therapy. The participants were recruited through internal announcements to the student population at the University of Bergen, Norway by email and posters during the fall and spring 2014. Initially 56 participants were recruited, but due to poor data quality on the resting vmHRV measures, three participants were excluded from further analysis. Thus, the sample consisted of 53 students (36 (68 %) female, mean age = 23.6 years, SD = 2.52). Of these, 34 participants wore monitors for 24 h, however due to poor data quality eight were excluded from further analysis. The subsample thus consisted of 26 participants, representative of the main sample with regard to the gender distribution, age range, and BMI (16 (62 %) women; mean age = 23.85; SD = 2.72).

Exclusion criteria were heart conditions, usage of sedative or psychoactive medication, and previous experience with mindfulness, i.e., attendance at mindfulness courses, retreats, or other kinds of formalized mindfulness instruction. The protocol was approved by the Regional Ethics Committee (South-East) and the participants gave written consent in accordance with the Helsinki declaration.

### Procedure

Upon arrival on the testing day, participants were given a detailed explanation of the tests they would undergo, but no information regarding hypotheses of the study. They were then assigned to a room in which they were asked to fill out a package of questionnaires, including the self-compassion scale and information about age, gender, and BMI. One at a time, they were asked to move to an experimental room, in order to record their heart rate with an electrocardiogram (ECG). After that, the 24-h monitors were attached. Participants were asked not to smoke, drink caffeine, or exercise 6 h prior to the experiment.

### Measures

#### Heart Rate Variability

The electrocardiogram (ECG) was recorded with a standard lead-II setup and digitized at 1000 Hz. The signal was obtained through an A/D converter (Biopac, MP36, Biopac system INC. Santa Barbara, CA) recorded with Biopac 4.0 BSL (Biopac Systems, Inc., Santa Barbara, CA). The data were collected at approximately the same time in the afternoon for all participants in order to control for circadian effects. Resting vmHRV was assessed during a 5-min period with the following instruction: “Give yourself some time to find a position that feels comfortable. See if you can breathe slowly, and relax as much as possible.”

Heart rate data were first checked manually for artifacts (electrode noise, movement, and extraordinary peaks) offline, before they were subjected to a vmHRV analysis with Kubios version 2.0 (Tarvainen et al. 2014). Low frequency was defined as the frequency band between 0.04 and 0.14 Hz, and high frequency was defined as 0.14–0.4 Hz. The applied measure was the root mean square of successive differences (RMSSD), measured in milliseconds. RMSSD is considered to be a valid measure of vmHRV (Li et al. 2009; Thayer and Sternberg 2006; Williams et al. 2015), and has a high trait specificity of 73 % (Bertsch et al. 2012), suggesting that the one-time assessment of RMSSD predominantly indicates a physiological trait measure (Williams et al. 2015). Moreover, RMSSD seems to be less affected by breathing than the high-frequency (HF) power (Penttilä et al. 2001). As participants were told to breathe naturally, RMSSD is probably the best index of vagally mediated heart rate variability in our sample. However, the applied RMSSD measure correlated highly with high-frequency (HF) power (*r* = .94, *p* > .001) in the main sample (*n* = 53).

For a subsample of 26 participants, 24-h inter-beat interval (IBI) data was also acquired using Actiheart monitors (Cambridge Neurotechnology, Cambridge, UK)—a device that has shown to give a reliable account of IBI data (Brage et al. 2005). The Actiheart recorder was placed horizontally below the apex of sternum, midway below the V1 and V2 positions using two adhesive Ag/AgCl ECG electrodes (T815 Dia. 55).The IBI data was transferred into a computer with the Actiheart commercial software (Actiheart software 2.132), and noisy and missing HR data was edited using the manufacturers algorithm (Cambridge Neurotechnology Ltd.; Brage et al. 2005). The complete IBI time series was subsequently inspected and remaining artifacts were manually removed.

All HRV data were subsequently subjected to a HRV analysis with Kubios version 2.0 (Tarvainen et al. 2014) from which root mean square of successive differences in R-R intervals (RMSSD) was calculated. Trend components were removed with the smoothness priors detrending method (*λ* = 500).

##### Self-Compassion Scale

The Self-Compassion Scale (SCS) (Neff 2003a) consists of 26 items loading on three positive and three negative subscales. The positive subscales are: self-kindness (for example: “I’m kind to myself when I’m experiencing suffering.”), common humanity (for example: “When I’m down and out, I remind myself that there are lots of other people in the world feeling like I am”), and mindfulness (for example: “When I fail at something important to me I try to keep things in perspective”). The negative subscales are: self-judgment (for example: “I’m disapproving and judgmental about my own flaws and inadequacies.”), isolation (for example: “When I’m feeling down, I tend to feel like most other people are probably happier than I am”), and over-identification (for example: “When I’m feeling down I tend to obsess and fixate on everything that’s wrong”). Items are rated on a five-point Likert-type scale from 1 (“almost never”) to 5 (“almost always”). High scores on the positive subscales and low scores on the negative subscales reflects an overall high level of self-compassion.

The SCS has shown good reliability and cross-cultural validity (Neff et al. 2008). We used a Norwegian translation of the Self-Compassion Scale (Dundas et al. 2015). In the current study, the level of self-compassion ranged from 1.31 to 4.27, with a mean level of 2.78 (SD = 0.83).

##### Rumination

The 12-item Rumination subscale of the Rumination-Reflection Questionnaire (RRQ-Rum) (Trapnell and Campbell 1999) was used. Example items are “My attention is often focused on aspects of myself I wish I’d stop thinking about” and “I often find myself re-evaluating something I have done.” Answers are rated on a five-point Likert-type scale ranging from 1 (“strongly disagree”) to 5 (“strongly agree”). The RRQ-Rum scale has been reported to have a high internal reliability (Cronbach’s alpha = .91; (Verplanken et al. 2007). The level of RRQ-Rum in the present study ranged from 14.0 to 57.0, with a mean level of 43.71 (SD = 10.58).

##### Trait Anxiety

Trait anxiety (STAI) was measured using the Trait scale of the State-Trait Anxiety Inventory (STAI; Spielberger 1983), consisting of 20 items. Examples of items are “I worry too much over something that really doesn’t matter” and “I have disturbing thoughts.” Scores are rated on a four point Likert scale ranging from 1 (“almost never”) to 4 (“almost always”). The Trait scale of the STAI has shown excellent internal consistency (average α < .89) and test-retest reliability (average *r* = .88; Barnes et al. 2002). The level of trait anxiety in the current study ranged from 20 to 69 with a mean of 43.75 (SD = 12.58), thus overlapping with other study samples of university students both regarding range (Williams et al. 2015) and mean scores (Andrade et al. 2001).

### Statistical Analysis

All HRV measures were log transformed in order to approximate a normal distribution. The data were analyzed statistically using the Statistical Package for the Social Sciences version 22.0 (SPSS; IBM Corp. 2011). A multiple hierarchical linear regression analysis was computed including the vmHRV as the outcome variable and the SCS total score as a predictor. In the first step of this hierarchical linear regression analysis, the possible confounding variables of age, sex, and BMI were entered as covariates, and in the second step the SCS total score was entered as a predictor (i.e., with *F* analysis of change in explained variance from step 1 to step 2). Further, we conducted a follow up analysis of how the SCS total score predicted vmHRV when adding covariates of trait anxiety and rumination in the first step of the multiple hierarchical linear regression analysis together with the covariates of age, sex, and BMI. To avoid problems with multicollinearity when controlling for level of trait anxiety and rumination, posed by the expected high correlation between trait self-compassion and trait anxiety/rumination, we entered residual scores where the variance in the symptom scores of trait anxiety and rumination that was explained by the self-compassion scale score were extracted (i.e., residual scores of trait anxiety and of rumination: the variance explained by the SCS total score was extracted in linear regression analyses run prior to this main multiple hierarchical linear regression analysis). Bivariate correlational analyses were conducted to show the relationship between level of vmHRV and the total score of SCS, the subscales of SCS, and rumination and trait anxiety. To ecologically validate the predictive effect of SCS total score on resting HRV, we ran a partial and bivariate correlational analysis in a subsample (*n* = 26) between SCS total score and a 24-h vmHRV measure. Similar to the 5-min resting measurements, the applied measure of 24-h vmHRV was also the RMSSD.

There were no outliers in the current sample defined by a plus/minus 3 standard deviation threshold from the sample mean. We used an alpha level of 5 % (*p* < .05) as a threshold for significant effects. Missing item scores were replaced by sample mean for each item. Five participants had some missing items scores, and a total of six item scores were missing.

## Results

The results showed that the SCS total score explained 12 percent of the variance of the vmHRV after controlling for the effects of the covariates of age, gender, and BMI in the analysis. These covariates did not contribute significantly to explain the variance of vmHRV either in the first or the second step of the analysis (see Table 2, model 1). Results were also significant using HF and NN50 as outcome measures of vmHRV (see supplementary tables).

**Table 2:** Hierarchical regression analyses of the relationship between vmHRV and self-compassion

Total sample	Model	Step	Predictor	*R* ^2^	*∆R* ^2^	*df*	*∆F*	*β* step 2
(*n* = 53)	Model 1	1	Age	.03	.03	3/49	.55	.02
			Gender					−.24
			BMI					−.06
		2	Self-compassion	.15	.12	1/48	6.56	.37*
	Model 2	1	Age	.04	.04	5/47	.42	.03
			Gender					−.21
			BMI					−.06
			STAI-T residual					−.03
			RRQ residual					.09
		2	Self-compassion	.16	.11	1/46	6.14	.36*

STAI-T residual—the variance explained by the SCS total score is extracted from the STAI-T score in a linear regression analyses with the STAI-T as an outcome variable and the SCS total score as a predictor. RRQ residual—the variance explained by the SCS total score is extracted from the RRQ score in a linear regression analyses with the RRQ as an outcome variable and the SCS total score as a predictor. Age, gender, and BMI were included as covariates in the first step of the hierarchical regression analysis. In the second model, the residual scores of trait anxiety and rumination were also included in the first step

*p = .02

To control for the possible confounding effect of higher levels of trait anxiety and rumination in the positive prediction of the SCS total score on the vmHRV, we repeated the same multiple hierarchical linear regression analysis by adding residual scores of trait anxiety and rumination as covariates in addition to age, gender, and BMI in the first step of the analysis. Still, the SCS total score explained 11 % of the variance of the vmHRV after controlling for the effect of these covariates on vmHRV, and SCS was the only significant contributor to explain the variance of the vmHRV in the second step of the analysis (see Table 2, model 2).

Bivariate correlational analyses showed that higher levels of the SCS total score related negatively with higher levels of trait anxiety and rumination (see Table 3). Furthermore, the positive SCS subscale scores were inversely correlated with trait anxiety and rumination, whereas the negative SCS subscales were positively correlated with trait anxiety and rumination. The trait anxiety residual score (in which the variance that was explained by the SCS total score in the trait anxiety score was extracted) correlated only with the trait anxiety score and the rumination residual score. The rumination residual score (in which the variance that was explained by the SCS total score was extracted) correlated with the rumination score, the self-compassion subscale of common humanity, as well as the trait anxiety score and the residual score of trait anxiety.

**Table 3:** Bivariate correlations between vmHRV, SCS, RRQ, and STAI

	1	2	3	4	5	6	7	8	9	10	11	12
1. vmHRV (rest)		.31*	.27***	.16	.21	−.28*	−.30*	−.29*	−.14	.26***	.13	−.01
2. SCS total			.83**	.76**	.80**	.84**	−.86**	−.85**	−.73**	−.80 **	.00	.03
3. Self-kindness				.58**	.63**	−.86**	−.54**	−.57**	−.59**	−.54**	.01	.22
4. Common humanity					.61**	−.56**	−.64**	−.49**	−.40**	−.61**	.27*	.08
5. Mindfulness						−.52**	−.57**	−.71**	−.55**	−.61**	.04	.03
6. Self-judgment							.61**	.61**	.66**	−.58**	.06	−.19
7. Isolation								.79**	.62**	.78**	−.01	.14
8. Over-identification									−.77**	.77**	.21	.15
9. RRQ (rumination)										.74**	.68**	.22
10. STAI-T (anxiety)											.35**	.57**
11. RRQ residual												.35**
12. STAI-T residual												
Subsample (*n* = 26)												
24-h vmHRV	.50*	.30	.30	−.05	.15	−.20	−.38	−.31	−.26	−.27	−.02	−.07

*N* = 53. RRQ residual—the variance explained by the SCS total score is extracted from the RRQ score in a linear regression analyses with the RRQ as an outcome variable and the SCS total score as a predictor. STAI-T residual—the variance explained by the SCS total score is extracted from the STAI-T score in a linear regression analyses with the STAI-T as an outcome variable and the SCS total score as a predictor

*p < .05; **p < .01; ***p = .06

Bivariate correlational analyses further showed that higher levels of vmHRV correlated significantly with higher SCS total scale scores, and that higher vmHRV correlated with lower scores on the negative subscales of self-judgment, isolation, and over-identification. The positive self-compassion subscale self-kindness was marginally significant (*p* = .06). Higher levels of vmHRV was marginally correlated with trait anxiety (*p* = .06), but did not correlate with rumination. Neither the trait anxiety residual score nor the rumination residual score was found to correlate with vmHRV.

**Fig. 1:**
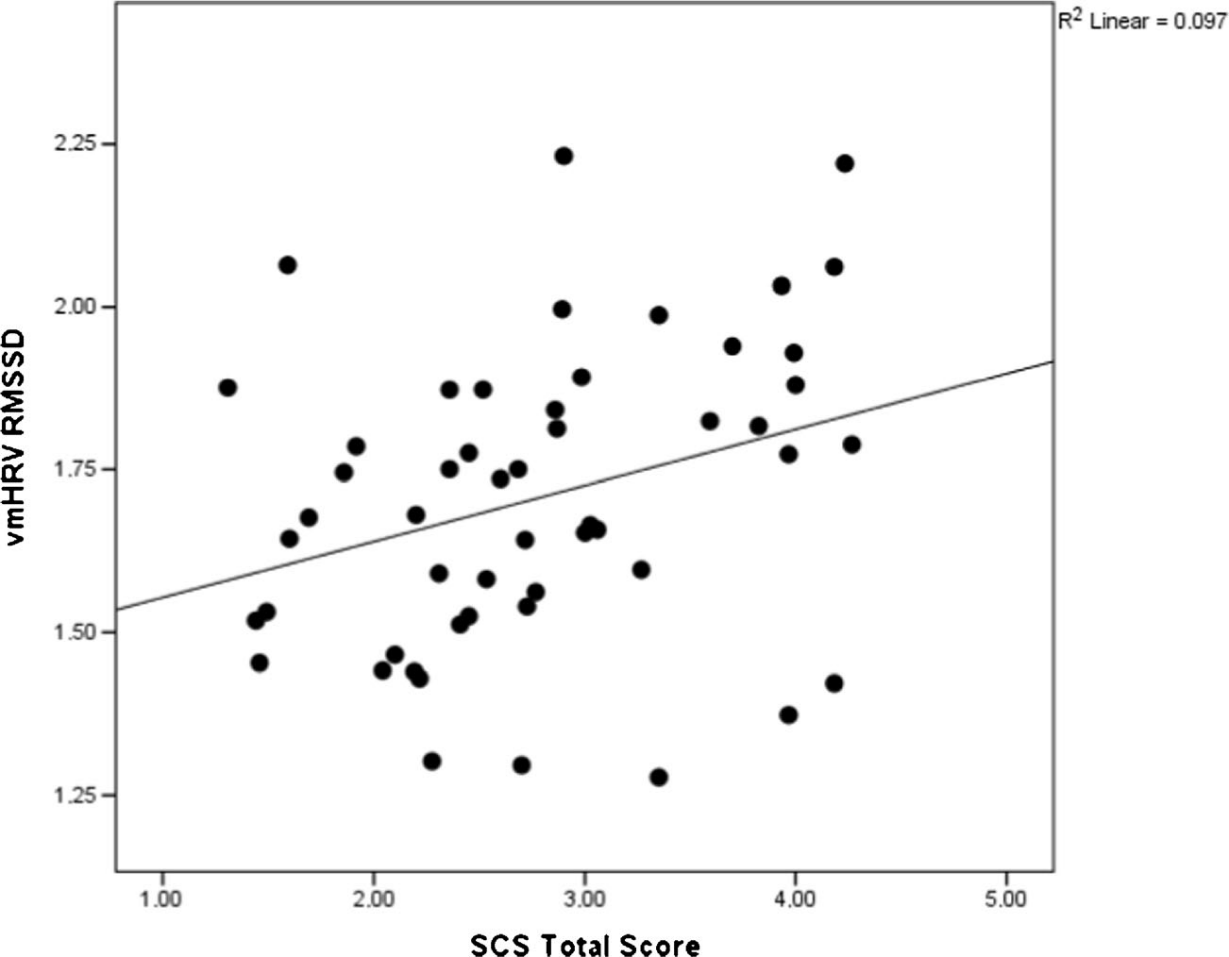
Scatterplot on the relation between self-compassion and vmHRV

A partial correlation analysis, controlling for the covariates of age, gender, and BMI, showed that the SCS total score correlated significantly also with the 24-h vmHRV (*df* = 21; *r* = .50; *p* < .02), showing a similar level of association as with the 5-min resting vmHRV in this subsample (*df* = 21; *r* = .52; *p* = .01). Bivariate correlation analyses showed that the 24-h vmHRV associated moderately with the 5-min resting vmHRV. Further, these correlation analyses showed that the SCS total score correlated with the 24-h vmHRV at the same level as the SCS total score correlated with the 5-min resting vmHRV in the bivariate correlational analyses conducted in the total sample (Fig. 1).

## Discussion

The aim of the current study was to investigate the relation between trait self-compassion and vmHRV (i.e., RMSSD) in a group of young, healthy adults. Our findings suggest that higher levels of trait self-compassion predicts a better ability to physiologically adapt emotional responses after contextual conditions in that higher SCS total scores predicted higher levels of vmHRV. These findings were ecologically validated with a 24-h measure of vmHRV, acquired from a subsample of the participants, confirming the positive correlation between high trait self-compassion and higher vmHRV. As expected, being more self-compassionate also associated with lower levels of trait anxiety and rumination, but the positive association between self-compassion and vmHRV could not be explained by trait anxiety or rumination. These results indicate that self-compassionate individuals have more adaptive emotion regulation abilities and less emotional stress reactions.

Despite the large base of studies reporting positive effects of self-compassion on mental health (e.g., Neff et al. 2005; Neff et al. 2007a), this is as far as we know the first study to show an association between high trait self-compassion and high vmHRV. However, our findings correspond to previous studies showing higher state-induced self-compassion to predict higher vmHRV (Arch et al. 2014; Kok et al. 2013; Rockliff et al. 2008), and previous studies showing higher induced (Pace et al. 2009) and trait self-compassion (Breines et al. 2015; Breines et al. 2014) to predict other biological stress indexes than vmHRV. The finding that a better psychological emotion regulation strategy reflects an enhanced physiological emotional regulation in increased vagal inhibition of the heart is in accordance with the theories of Porges (2007) and Thayer and Lane (2000). Self-compassion as an emotion regulation strategy thus seems to allow people to be more flexible when facing situations that can elicit stress reactions.

Self-compassion can be understood to effect multiple levels of emotion regulation, such as situation selection, situation modification, attention, cognitive change, and response modulation as conceptualized by Gross (2014). On the level of attention, self-compassionate individuals may be less attentive to threat cues and more attentive to soothing cues, which serves to reduce emotional arousal. Also, having a broader attentional focus in different situations may help to create a psychological distance between experiences and self-value, so that one is able to observe and reflect in an emotional situation, rather than automatically reacting on the first spontaneous emotional impulse. On the level of cognitive change, self-compassionate individuals may have more resources available to cognitively moderate their view of stressful situations. This is because self-compassion may have a soothing effect on the limbic system, thereby liberating prefrontal resources and allowing for more explicit (i.e., more conscious and effortful) regulation such as cognitive reappraisal. Consequently, self-compassionate individuals may open to a more balanced views of stressful situations and engage in more supportive and soothing self-talk than less self-compassionate individuals.

This emotion regulatory capacity of self-compassion was further supported in that self-compassion reflected lower levels of trait anxiety and rumination. Thus, individuals low in self-compassion and high in self-criticism seem to have reduced capacity to modulate emotions. This may manifest in a bias toward threat cues (attention) and negative interpretations (cognitive change) of situations, in which they tell themselves that it is their fault that they feel bad, or that they always make mistakes. These stress reactions through negative self-talk serves to activate the body’s stress response and deactivate the body’s self-soothing system (Gilbert 2005). This, in turn, leads to lower vmHRV, due to vagal withdrawal (Porges 2007; Thayer and Lane 2000). Indeed, the findings in the current study showed that the self-critical SCS subscales all predicted lower vmHRV, which points to a higher level of emotional stress reactions in everyday life. These negative SCS subscales also correlated with higher levels of anxiety and rumination.

Self-compassion as a trait covers both compassionate and uncompassionate behavior toward oneself, as represented by the positive and negative subscales, and recently Neff (2016) has emphasized that the lack of self-compassion is as important as the presence of it. We found that it was mainly the negative SCS subscales that showed to associate with vmHRV, although the positive SCS subscales were in the predicted direction, and the positive subscale of self-kindness was marginally significant (*p* = .06). The negative SCS subscales reflect a self-critical attitude toward oneself, feeling alone and isolated with one’s thoughts and emotions, as well as identifying highly with them. This is interesting since isolation has been subject to much less research attention than self-criticism and over-identification (Neff 2016), which have long been recognized as important vulnerability factors in psychopathology (Blatt et al. 1982; Germer 2009; Gilbert and Procter 2006). There is evidence that early family experiences and insecure attachment make some individuals more vulnerable to develop high levels of self-criticism (Gilbert et al. 2004). Such experiences may also lead to altered cardiovascular responses to stress (Luecken et al. 2005), as the first living years represent an important period for the myelination process of the vagus nerve (Porges 2007). Thus it is possible that the negative effect on vmHRV of being self-critical, self-isolated, and self-absorbed is stronger than the positive effect of being self-supporting. However, it is important to note that the positive subscales also showed a tendency to associate with vmHRV. In particular, the positive subscale of self-kindness associated almost as strongly with vmHRV as did the negative subscales. This means that the positive subscales also contributed to the total SCS score predictions of high vmHRV, which is also supported in the high correlation between all the SCS subscales and the total SCS score.

There may be many reasons why individuals differ in their levels of self-compassion. The quality of attachment with significant caregivers in childhood may be one such reason, in addition to the amount of stress and negative life events and the level of self-regulative abilities (Gilbert 2005). Being more self-compassionate can therefore be both a result of an innate trait and/or a trait that has been nurtured. In our study, we predicted higher self-compassion to lead to higher vmHRV based on theories of self-compassion as an emotion regulation strategy (Neff 2003b), and on this basis we treated self-compassion as a trait. However, with a cross-sectional study design, we cannot draw certain conclusions about causality (see Williams et al. 2015) (see Williams et al. 2015). Thus, the previous findings that state-induced self-compassion predicts higher vmHRV (Arch et al. 2014; Rockliff et al. 2008) (Arch et al. 2014; Rockliff et al. 2008), support our hypothesis that it is higher self-compassion that predicts higher vmHRV.

Also, as the diagnosis of anxiety and the tendency to ruminate are shown to associate with lower vmHRV (e.g., Gorman and Sloan 2000; Williams et al. 2015; Woody et al. 2014), we wanted to ensure that the prediction of high self-compassion on high vmHRV was not better explained by lower levels of anxiety or rumination. We found that higher levels of anxiety together with rumination were strongly correlated with low self-compassion, which may be due to them capturing a wider aspect of emotional regulation than just emotional stress (Andrade et al. 2001; Caci et al. 2003). However, of these self-reported forms of emotional stress, it was only trait anxiety that marginally significantly correlated with vmHRV. This may seem surprising, given that previous research has found anxiety and rumination to significantly associate with vmHRV (Williams et al. 2015). Noteworthy is that we found the same correlation level as did these authors (trait anxiety −.27), however, the significance level appeared different; a differential effect probably due to a larger sample size in the study of Williams et al. (2015). In the prediction analyses, low trait anxiety or rumination did not better explain high vmHRV than did high trait self-compassion. This was further demonstrated in that residual trait anxiety and rumination scores consisting of the variance not explained by self-compassion did not correlate with vmHRV, but correlated strongly with the trait anxiety score and rumination score, respectively.

The present study has several limitations. First, the sample consisted of a relatively small number of well-functioning, healthy young adults, which may reduce generalizability of findings to other populations and age ranges. Second, the present study used a cross-sectional design, thus the question of causality remains unknown. Although higher levels of self-compassion may protect against lower levels of vmHRV, it is also possible that a person’s level of vmHRV influences his or her ability to be self-compassionate. Thus, future research would benefit from using longitudinal designs to examine the effects of changing self-compassion levels on vmHRV.

The present results may have several clinical implications. Our results contribute to a growing awareness of self-compassion as an adaptive emotion regulation strategy, and indicate that self-compassion predicts flexible physiological responding in potentially stressful situations. Developing higher self-compassion may thus help individuals to more flexibly adapt their emotions and physiological responses in different situations. Indeed, there is today a growing focus on training the ability to be more self-compassionate, for example, through mindfulness training such as MBCT (Kuyken et al. 2010), a specific self-compassion training program (Mindful Self-Compassion program; Neff and Germer 2013), or through compassion-focused therapy (Gilbert 2010). Further, as reduced vmHRV is a risk factor for cardiovascular disease and all-cause mortality (Thayer and Lane 2007) our finding that self-compassion predicts vmHRV may have implications for physical health. Increasing self-compassion may thus not only have positive effects on psychological health, but also on physiological health, through enhanced vagal activation.

## Electronic supplementary material

Below is the link to the electronic supplementary material.

ESM 1 (DOCX 19 kb)
