# Supplementary material for: Trait Self-Compassion Reflects Emotional Flexibility Through an Association with High Vagally Mediated Heart Rate Variability
Source: Mindfulness (N Y). 2016 Jun 2;7(5):1103–13. doi: 10.1007/s12671-016-0549-1 (PMC5010618; doi:10.1007/s12671-016-0549-1)
Supplement: Supplementary file 1 — (DOCX 19 kb) [file 12671_2016_549_MOESM1_ESM.docx]

**Supplementary tables**

**Supplementary table 1: HF and self-compassion**

Note: *= p=.05; **=p=.01. HF= High frequency heart rate variability. Age, gender and BMI were included as covariates in the first step of the hierarchical regression analysis.

**Supplementary table 2: NN50 and self-compassion**

Note: *= p=.05; **=p=.02; ***=p=.01. NN50= Number of pairs of adjacent NN intervals differing by more than 50 ms. Age, gender and BMI were included as covariates in the first step of the hierarchical regression analysis.
